# Recurrent Hypokalemia as an Unusual Presentation for Metastatic Adrenocortical Cancer

**DOI:** 10.7759/cureus.25954

**Published:** 2022-06-15

**Authors:** Naomi Mirza, Erinie Mekheal, Brooke E Kania, Vinod Kumar, Michael Maroules

**Affiliations:** 1 Internal Medicine, St. Joseph's Regional Medical Center, Paterson, USA; 2 Oncology, St. Joseph Regional Medical Center, Paterson, USA; 3 Hematology and Oncology, St. Joseph's University Medical Center, Paterson, USA

**Keywords:** refractory hypokalemia, hypercortisolism, hypokalemia, metastatic adrenal cancer, adrenocortical tumors

## Abstract

Adrenocortical cancer is a rare malignancy of the endocrine system. Therefore, when this malignancy presents with metastatic disease, this leads to further difficulties in management. Due to the rare and ambiguous nature of this malignancy, diagnosis is generally made at later stages, with limited options for patients. Symptoms may include weight gain/loss, muscle weakness, abdominal discomfort/bloating, hyperglycemia, hypertension, electrolyte imbalance, hirsutism and virilization in females, gynecomastia and hypogonadism in males. Due to the variety of conditions presenting with one or more of these symptoms, diagnosis can be difficult. Many adrenocortical tumors, malignant and benign, are usually detected incidentally on imaging performed for evaluation of another condition, also known as “incidentalomas.” Here, we present a rare case of metastatic adrenocortical malignancy in a 56-year-old female patient who presented with isolated recurrent episodes of hypokalemia.

## Introduction

Adrenocortical cancer represents a rare malignancy (approximately 0.7-2 cases per million per year) found to be more common in women, with a higher incidence in both childhood (from patients 1-6 years old) and during the fourth to fifth decades of life [1,2]. Adrenocortical malignancies are often associated with familial hereditary syndromes such as multiple endocrine neoplasia type 1, Li-Fraumeni syndrome, Beckwith-Wiedemann syndrome, and Carney complex. However, sporadic adrenocortical carcinoma is a heterogeneous neoplasm with poorly understood pathogenesis [2]. We present a case of a patient diagnosed with adrenocortical cancer found to have complications of persistent hypokalemia, further exemplifying the challenges associated with this rare disease and the rapidly progressive nature of this malignancy.

## Case presentation

A 56-year-old female with a past medical history significant for benign essential hypertension, morbid obesity, a recent history of gastric bypass surgery with significant weight loss, and otosclerosis status post cochlear implantation presented to the Emergency Department (ED) with a referral from her primary care physician (PCP) due to generalized weakness and recurrent episodes of refractory hypokalemia over a four-month period.

Four months prior to presentation, the patient experienced bilateral lower extremity weakness that was thought to be secondary to nutritional deficiencies from her recent bariatric surgery. Initial outpatient workup revealed isolated mild hypokalemia (K of 3.0 mEq/L with a normal reference range of 3.5-5), with no other abnormalities in electrolytes and sufficient vitamin levels. Two months later, the patient experienced a similar episode of lower extremity weakness involving the proximal muscles that caused significant dysfunction in her daily activities and frequent falls. At that time, complete metabolic panel (CMP), creatinine phosphokinase (CPK), erythrocyte sedimentation rate (ESR), and antinuclear antibodies (ANA) were ordered and were within normal limits, except for a K of 2.3 mEq/L. the patient was started on daily potassium supplementation that was titrated up to 60 milliequivalents of oral supplements daily. A few days prior to her presentation, she experienced bilateral lower extremity swelling, facial swelling, hirsutism, and elevated systolic blood pressure ranging between 130 and 155 mmHg. Subsequent outpatient workup revealed low ACTH levels (<5; reference range 7.2 to 63.3 pg/mL), normal renin activity (reference range 0.167-5.380 ng/mL/hr) and aldosterone levels (<4; reference range 0-30 ng/dL), consistent with hypercortisolism.

The patient reported a positive family history of bladder and uterine cancer in her mother with subsequent lung metastasis. She denied a history of multiple endocrine neoplasia 1, Li-Fraumeni syndrome, Beckwith-Wiedemann syndrome, or Carney complex. She is a former smoker who quit 24 years ago, with a smoking history of one-half pack per day for seven years. She denied any alcohol intake or recreational drug use.

**Figure 1:**
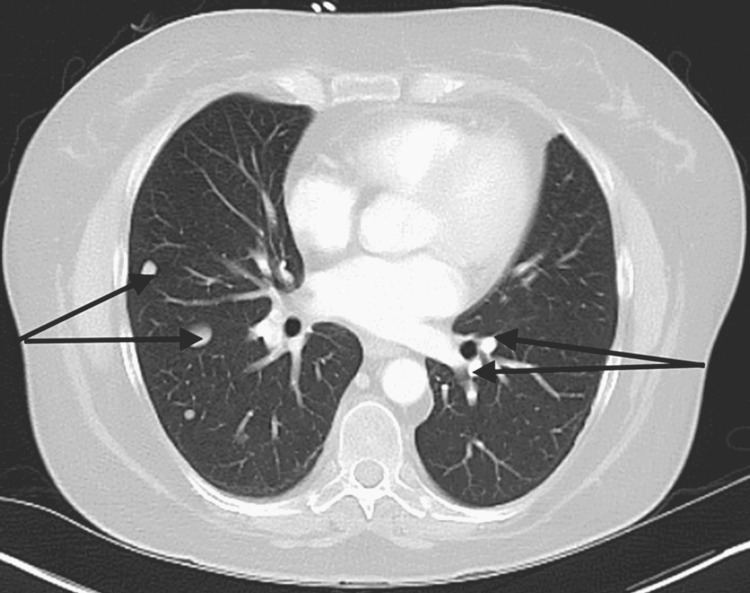
Computer tomography of chest axial view Computer tomography of chest showing multiple bilateral pulmonary nodules noted (black arrows) largest 1.5 cm in right lower lobe. Multiple bilateral pulmonary nodules were also noted concerning metastases. There were filling defects noted in the right lower lobe compatible with pulmonary emboli.

**Figure 2:**
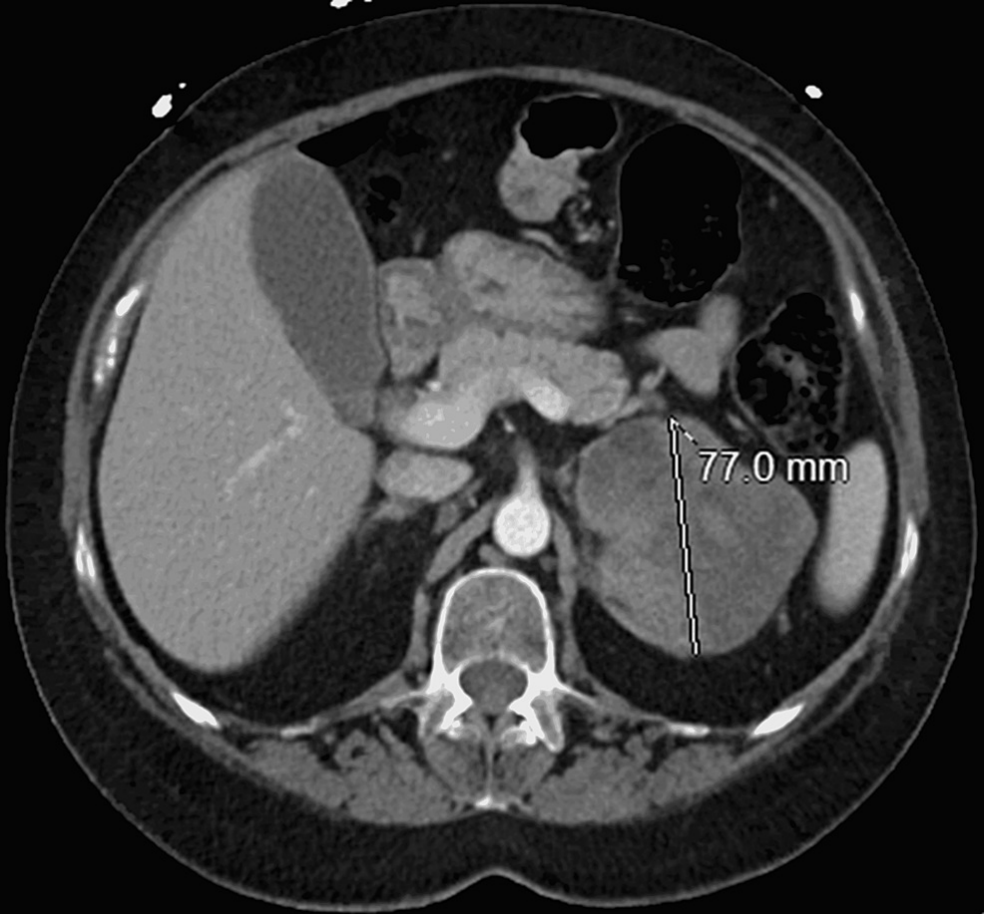
Computed tomography image of the abdomen showing adrenal mass on axial plane Image showing heterogenous 7.7 cm lesion (white line measure) appearing to be arising from left adrenal glands in contact with the upper pole of left kidney with areas of hypodensity suggesting necrosis.

**Figure 3:**
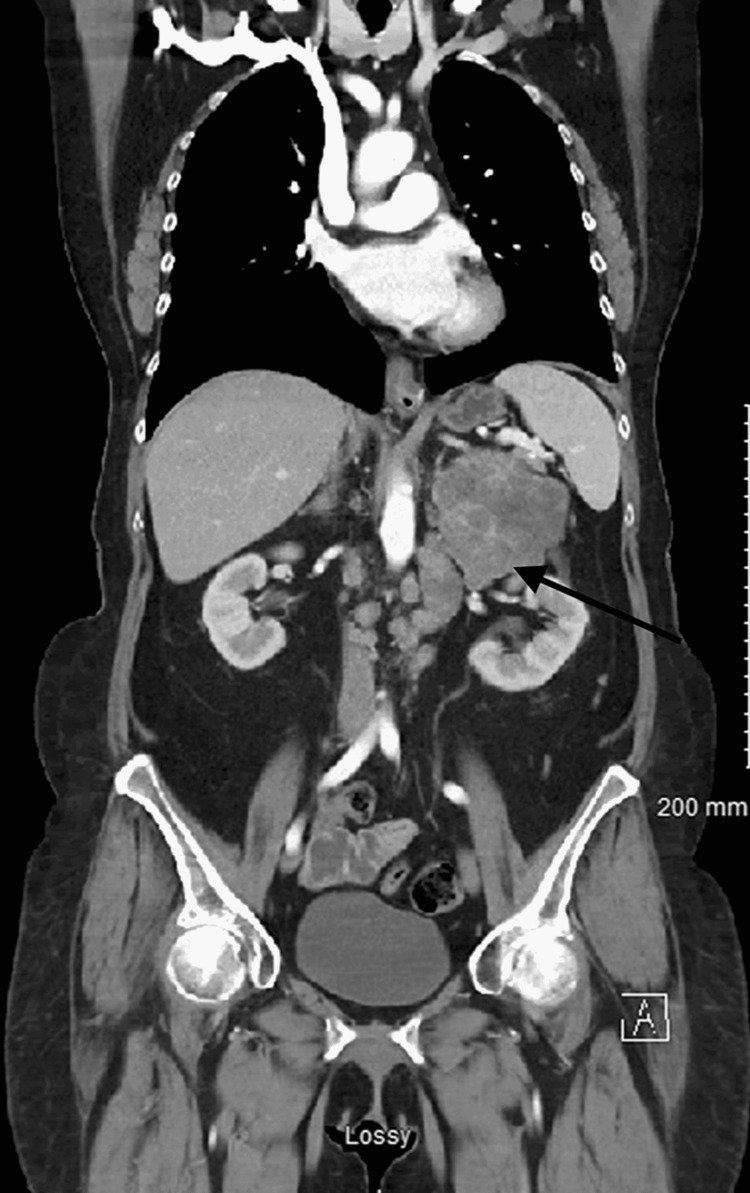
Computer tomography image of chest/abdomen/pelvis showing adrenal mass on coronal plane Image showing heterogenous 7.7 cm lesion in length (black arrow) appearing to be arising from left adrenal glands in contact with the upper pole of left kidney with areas of hypodensity suggesting necrosis.

In the ED on examination, the patient was not in acute distress. She was awake, alert, oriented, and obese. The cardiopulmonary examination was unremarkable. Her abdomen was distended and remarkable for striae. The facial examination was significant facial hair (hirsute appearance) and moon facies (facial swelling or roundness, as per the patient this had developed recently). Neurological examination was unremarkable. Initial laboratory tests in the ED were within normal levels except for hypokalemia, and metabolic alkalosis (Table 1). The patient was admitted for treatment of refractory hypokalemia and further workup for the underlying causes with possible hypercortisolism as our main working diagnosis as per the guidance of her PCP. Subsequently, biochemical laboratory tests have confirmed a diagnosis of Cushing syndrome (Table 2). Imaging with chest x-ray revealed multiple nodular opacities in the right lower lung zones, with the largest measuring 11 mm, and right upper lung zone with the largest measuring 8 mm. Additional several smaller lung nodules were visualized in the left lung. CT chest abdomen and pelvis was performed for further evaluation of these lesions and were significant for an 8 cm solid enhancing left adrenal mass with large retroperitoneal lymph nodes concerning underlying malignancy and bilateral pulmonary nodules with findings suggestive of metastasis, in addition to a filling defect noted in the RLL compatible with pulmonary emboli (Figures 1-3).

**Table 1:** Basic metabolic panel on admission

Electrolytes	Level	Reference range
Sodium	138 mEq/L	135-145
Potassium	2.7 mEq/L	3.5-5.0
Chloride	97 mEq/L	98-107
Bicarbonate	42 mEq/L	21-31
Creatinine	0.6 mg/dL	0.6-1.3
Glucose	99 mg/dL	70-110
Calcium	9.1 mg/dL	8.6-10.3
Magnesium	2.2 mg/dL	1.7-2.5

**Table 2:** Biochemical laboratory tests: hormone levels mcg/24 hr = micrograms in 24 hours; ACTH = Adrenocorticotropic hormone; 17-OH progesterone = 17-hydroxyprogesterone; DHEA -sulfate = Dehydroepiandrosterone (DHEA) sulfate. Reference ranges are provided for females age > 30.

Laboratory Test	Levels	Reference range
24 hour cortisol urine	1194 mcg/24h	6-42
AM cortisol	50 mcg/dL	8.7-22.4
ACTH	<5 pg/mL	7.2-63.3
Metanephrine, free	<0.2 mcg/L	36-209
Normetanephrines, free	<0.2 mcg/L	131-612
Free testosterone	3.66 pg/mL	0.4-6.7
Androstenedione	302 pg/mL	41-262
Estradiol	114.9 pg/mL	Variable, post-menopausal female reference range <6-54.7 pg/mL
17-OH Progesterone	326 ng/dL	Variable
DHEA-sulfate	634 mcg/dL	41.2-243.7

Endocrinology, Oncology, and Surgery teams were consulted, who recommended biopsy of the lung nodules, as well as the retroperitoneal lymph nodes to identify the origin of the possible cancer. In preparation for surgery and biopsy, anticoagulation for possible right lower lobe pulmonary embolism was withheld. Fine needle aspiration and core biopsy from the left adrenal mass was positive for malignancy, and pathology revealed adrenal cortical carcinoma. CT-guided core biopsy from three enlarged left para-aortic lymph nodes revealed highly atypical cells with mild to moderate atypia, suspicious for malignancy with areas of focal necrosis. Final cytological analysis from the lymph node biopsies revealed malignant cells compatible with adrenal cortical carcinoma as per immunohistochemistry (IHC) staining pattern and positive Weiss criteria (at least three out of nine Weiss Criteria required for diagnosis). Additionally, cells were positive for Inhibin A, confirming the final diagnosis. The patient’s right lung mass biopsy showed metastatic epithelial neoplasm with IHC staining suggestive of adrenal origin. The patient was ultimately transferred to a specialized cancer treatment facility for higher level of care. She was started on Enoxaparin at anticoagulation dose for the right lower lobe emboli. The decision was made to treat her with a combination of Metyrapone and Hydrocortisone, with subsequent mild clinical improvement. 

Unfortunately, the patient’s hospital course was complicated by interval development of a large retroperitoneal hematoma. Embolization was attempted by interventional radiology but was not successful as no clear source of bleed was found. It was not thought that the source of bleeding was from the primary tumor. Anticoagulation was withheld and an IVC filter was placed. The patient became hemodynamically stable with significant clinical improvement. She was discharged with a plan to initiate Mitotane and systemic chemotherapy. 

Following discharge, the patient had several ED visits due to episodes of severe hypokalemia with a syncopal episode for which she received intravenous potassium chloride. Eventually, the patient was started on systemic chemotherapy with a combination of Doxorubicin, Etoposide and Cisplatin for 28-day cycles with the plan to continue treatment for a total of six cycles. She continues to receive oral potassium supplementation for hypokalemia, in addition to Metyrapone and Hydrocortisone for management of her hypercortisolism, with plans to transition to Mitotane if Metyrapone fails. Due to her overall poor functional status, presence of other complications including a pulmonary embolism, and retroperitoneal bleed, the patient remains a poor candidate for surgery, with an average life expectancy possibly ranging from from several months to one to two years.

## Discussion

Recurrent or persistent hypokalemia is one of the rare manifestations of primary cancer. The most common causes of recurrent hypokalemia are renal tubular injury due to chemotherapy such as Cisplatin, gastrointestinal loss due to vomiting or diarrhea, and/or malabsorption due to intestinal resection for cancer treatment [3]. Isolated hypokalemia should raise concern for hypercortisolism secondary to an underlying corticosteroid or ACTH secreting tumor and should, when appropriate, warrant imaging.

This case represents the challenges associated with diagnosing and treating adrenocortical cancer. It also highlights how rapidly this may progress and the unfortunate poor prognosis in patients with adrenocortical cancers. Although the localized disease can be treated with surgery alone, unfortunately, about 80% of patients will experience local or distant recurrence after complete surgical resection of the adrenal tumor [4]. Cytoreductive debulking surgery may benefit patients with severe hormonal excess due to multiple metastatic lesions when medical management fails [5]. It is important to note that definitive therapy requires a broad approach involving multidisciplinary teams including Endocrinology, Surgery, Radiation, and Medical Oncology.

Medical treatment for adrenocortical malignancies revolves around symptom management from hormone excess. It may include Metyrapone which is a glucocorticoid synthesis inhibitor, or Mitotane which has a direct adrenal cortex cytotoxic effect causing permanent adrenal atrophy [2, 6]. Both Mitotane and Metyrapone are paired with supplemental Hydrocortisone to prevent adrenal crisis. Mitotane may be preferred for patients with advanced disease, such as ours, patients with extensive metastatic disease who may not be candidates for debulking surgery or patients who have failed surgical resection. Interestingly Mitotane, which is the only drug approved for adrenocortical carcinoma for both adjuvant therapy and advanced disease, has not had a randomized controlled trial to prove its efficacy [7]. Cytotoxic chemotherapies are not well studied and have not proved to be efficacious. However, The First International Randomized Trial in Locally Advanced and Metastatic Adrenocortical Carcinoma Treatment (FIRM ACT trial) elaborated that Mitotane in combination with Etoposide, Doxorubicin, and Cisplatin show better response rates and longer mean progression-free survival compared to Mitotane with Streptozotocin. Ultimately there was no difference in overall survival or risk of serious adverse events between both treatment groups [7]. Although radiation therapy may not be beneficial for primary or adjuvant treatment, palliative radiotherapy has been beneficial in treating symptomatic metastatic lesions. In preclinical models, mitotane has been studied as a radiosensitizer [2].

## Conclusions

In conclusion, recurrent or refractory electrolyte imbalance and other non-traditional signs of rare tumors with poor prognosis should be further evaluated. Chronic unexplained hypokalemia with other signs of hormonal over or underproduction, should raise suspicion for occult malignancy and may justify imaging for diagnosis. This case study confirms the importance of sound knowledge and experience with such tumors and the importance of a multidisciplinary approach to diagnosis and management to reduce mortality.
